# How do genes flow? Identifying potential dispersal mode for the semi-aquatic lichen *Dermatocarpon luridum* using spatial modelling and photobiont markers

**DOI:** 10.1186/s12898-020-00324-4

**Published:** 2020-10-15

**Authors:** Jennifer A. Doering, Tom Booth, Yolanda F. Wiersma, Michele D. Piercey-Normore

**Affiliations:** 1 Department of Biological Sciences, University of Manitoba, Winnipeg, MB R3T 2N2 Canada; 2 Department of Biology, Memorial University of Newfoundland, St. John’s, NL A1B 3X9 Canada; 3 Present Address: School of Science and Environment, Grenfell Campus, Memorial University of Newfoundland, Corner Brook, NL A2H 5G4 Canada

**Keywords:** *Dermatocarpon luridum*, *Diplosphaera chodatii*, Dispersal, Genetic variation, Lichenized alga, Semi-aquatic, Spatial modelling

## Abstract

**Background:**

Landscape genetics is an interdisciplinary field that combines tools and techniques from population genetics with the spatially explicit principles from landscape ecology. Spatial variation in genotypes is used to test hypotheses about how landscape pattern affects dispersal in a wide range of taxa. Lichens, symbiotic associations between mycobionts and photobionts, are an entity for which little is known about their dispersal mechanism. Our objective was to infer the dispersal mechanism in the semi-aquatic lichen *Dermatocarpon luridum* using spatial models and the spatial variation of the photobiont, *Diplosphaera chodatii*. We sequenced the ITS rDNA and the β-actin gene regions of the photobiont and mapped the haplotype spatial distribution in Payuk Lake. We subdivided Payuk Lake into subpopulations and applied four spatial models based on the topography and hydrology to infer the dispersal mechanism.

**Results:**

Genetic variation corresponded with the topography of the lake and the net flow of water through the waterbody. A lack of isolation-by-distance suggests high gene flow or dispersal within the lake. We infer the dispersal mechanism in *D. luridum* could either be by wind and/or water based on the haplotype spatial distribution of its photobiont using the ITS rDNA and β-actin markers.

**Conclusions:**

We inferred that the dispersal mechanism could be either wind and/or water dispersed due to the conflicting interpretations of our landscape hypotheses. This is the first study to use spatial modelling to infer dispersal in semi-aquatic lichens. The results of this study may help to understand lichen dispersal within aquatic landscapes, which can have implications in the conservation of rare or threatened lichens.

## Background

Landscape genetics is an interdisciplinary field that combines tools and techniques from population genetics with the spatially explicit principles from landscape ecology [32]. Spatial variation of genotypes has been used as a tool to test hypotheses about ecological processes [26]. One example of such an application is the use of spatial genetic variation to test hypotheses about how the physical landscape pattern affects dispersal [27]. Landscape genetics has been used to test hypotheses about dispersal for a wide range of taxa, including vascular plants (e.g., [3]), parasitic diseases (e.g., [44]), and even humans (e.g., [4]). Here we use landscape genetics to study the dispersal in a lichen symbiont. Given that many lichens consist of an algal partner that often has an aquatic habitat, we may be able to use landscape genetics to understand the dispersal of lichen. Landscape genetics have been used to study water-borne dispersal in lake organisms such as nematodes [12]. While lichen algae and free-living nematodes have very different life histories, the universality of genetic mechanisms across taxa and the success in applying landscape genetics for a wide range of species and systems suggests that landscape genetics may be a useful tool here.

The life cycle of most lichens, which are symbiotic associations between fungal (mycobionts) and photosynthetic (photobionts) partners, involve obligate associations for the fungal partner but facultative associations for the algal partner. Further complicating the life cycle is the ability to disperse through non-symbiotic and symbiotic methods. For many species of lichens, non-symbiotic methods include ascospore (sexual reproduction) and conidiospore (asexual reproduction) dispersal of the fungal component alone, which occurs independently of the photobiont. The germinating fungal spore must come into contact with a compatible photobiont (an alga or a cyanobacterium) in order for lichenization to occur. Symbiotic methods include thallus fragments or specialized propagules that contain both symbionts, which may grow into a lichen as soon as conditions are suitable. An examination of spatial variation in lichen symbionts must consider these complicating aspects of the symbiont life cycles but will likely yield valuable insights into symbiont population structure and lichen dispersal mechanisms.

Landscape genetics, which examine how geographic distances are related to similarities and differences in population genetic structure [47] could be an effective aid to understanding lichen dispersal mechanisms (but see [42]). Although work on landscape genetics of lichens has been limited, Fernández-Mendoza et al. [16] found that isolation by distance of the photobiont from temperate to Polar Regions could not account for different population structures in the same species of lichens and others have used the epiphytic lichen *Lobaria pulmonaria* as a model species for landscape genetic work [51, 54–56]. Walser et al. [51] found that there was separation of populations in the lichen-forming fungus, *Lobaria pulmonaria* (L.) Hoffm. due to glacial and postglacial histories among geographic locations, suggesting that physical features of the local landscapes and regional spatial scales are also needed to understand dispersal. Since population structure may not always correspond to geographical regions, Franscisco de Oliveira et al. [21] hypothesized that the photobiont may be more widely dispersed than the mycobiont. They also hypothesized that other factors such as fungal and algal genetic compatibility or environmental features may be influencing algal population structure [21]. A low level of population structure at a local level may indicate high levels of gene flow [57] or influences from genetic drift, sexual reproduction, historical processes, or dispersal vectors, such as water, wind, or animal dispersal.

Gene flow was previously studied for the photobiont (*Diplosphaera chodatii*) of a semi-aquatic lichen, *Dermatocarpon luridum,* between large geographic areas of different continents [20] showing that gene flow occurred mainly within continents and a low level between continents. Fontaine et al. [20] inferred isolation by distance but the geographic scale was too broad to make conclusions on gene flow within lakes of the same continent. Since *D*. *luridum* is a semi-aquatic lichen that grows on the rocks around the shoreline, it is periodically submerged in water throughout the summer and its life cycle, like other semi-aquatic lichens, is thought to benefit from submergence (COSEWIC [10, 11]). However, Fontaine et al. [20] did not take landscape ecology into account to investigate the dispersal mechanisms of water and/or wind vectored gene flow within a single lake. If the lichen alga, *Diplosphaera chodatii*, is dispersed by water (as co-dispersal of lichen fragments), the direction of gene flow would be expected to be same as the direction of water flow in the lake, though it may also be affected by the inflowing streams as has been shown in nematodes [12]. Larger source streams with more tributaries may yield higher numbers of haplotypes than smaller streams because of the greater potential for rock substrata necessary for lichen occurrence around the larger streams. If the lichenized alga within thallus fragments is dispersed by wind, the direction of gene flow would follow that of dominant wind patterns in the area. This may result in differences in the spatial and genetic variation of *D. chodatii* within *D. luridum* thalli around a single lake. Understanding how the lichenized alga may be spatially dispersed within a single lake can help to assess how similar lichens growing in aquatic environments may also disperse.

Many lichenized algae are also thought to be free living [1, 22]. *Diplosphaera chodatii* associates as the photobiont partner with *D. luridum* as well as with *Placynthiella dasaea* [40]. *Diplosphaera chodatii* has been observed to occur free-living in the soil [17, 31], on wood [25], and in extreme habitats such as deserts [18], but the ability for the free-living strains to lichenize has not been examined, and therefore it is unknown whether it could have an effect on the local population genetics of *D. luridum*. If additional free-living strains were brought in via upstream sources or run-off from cliffs, the increase in potential genetic variation could create unique population structure within the local environment. However, due to the slow growth of the lichen thallus, it is unlikely that any free-living *D. chodatii* could be incorporated into the thallus within a single growing season. Hence, in this study we focus only on the lichenized alga within thallus fragments as an indication of lichen fragment dispersal.

Here, we conduct a localized study of the population genetics of the photobiont (the lichenized alga *Diplosphaera chodatii*) of the semi-aquatic lichen *Dermatocarpon luridum*, growing within a thallus on rocks around Payuk Lake, a boreal lake in northern Manitoba, to assess whether spatial variation in the lichenized algal genetics can be used to make inferences about the lichen’s dispersal mechanisms and vectors.

## Results

### Evidence suggests that *D. chodatii* is free-living

Algal cultures from the rock scrapings showed a total of eight algal taxa (Additional file 1: Figure S1.2 and Table S2) isolated from rock surfaces surrounding *D. luridum* thalli. The first taxon was that of the photobiont, *Diplosphaera chodatii*. This species was characterized by its unicellular, ellipsoidal to spherical shape, ranging in size from 3–5 μm × 4–8 μm, and containing parietal chloroplasts (Additional file 1: Figure S1.2 and Table S2). Four other green algae were identified, and were all unicellular and spherical, either occurring singly or in a mucilaginous colony, ranging in size from 3–16.8 μm in diameter, and containing central or evenly interspersed chloroplasts (Additional file 1: Figure S1.2 and Table S2). There were two unidentified unicellular and spherical Chlorophyte green algae (Additional file 1: Figure S1.2 and Table S2), ranging from 4–5.4 μm × 4.4–5.5 μm in diameter. One of the unknown Chlorophytes had granular bodies present. Lastly, there was one unidentified cyanobacterium present with spherical cells arranged in a single trichome and heterocysts spaced throughout (Additional file 1: Figure S1.2 and Table S2). The presence of free-living *Diplosphaera chodatii* in the environmental sample was also supported by PCR (Additional file 1: Figure S1.3) where *Diplosphaera*-specific primers produced a single band at 750 bp in the same position as the ITS band produced in the lichenised *Diplosphaera* sample. However, definitive identification from an algal taxonomist and DNA sequencing of the PCR band would be needed to confirm our identification.

### Genetic diversity is contained within dominant haplotype networks

**Fig. 1:**
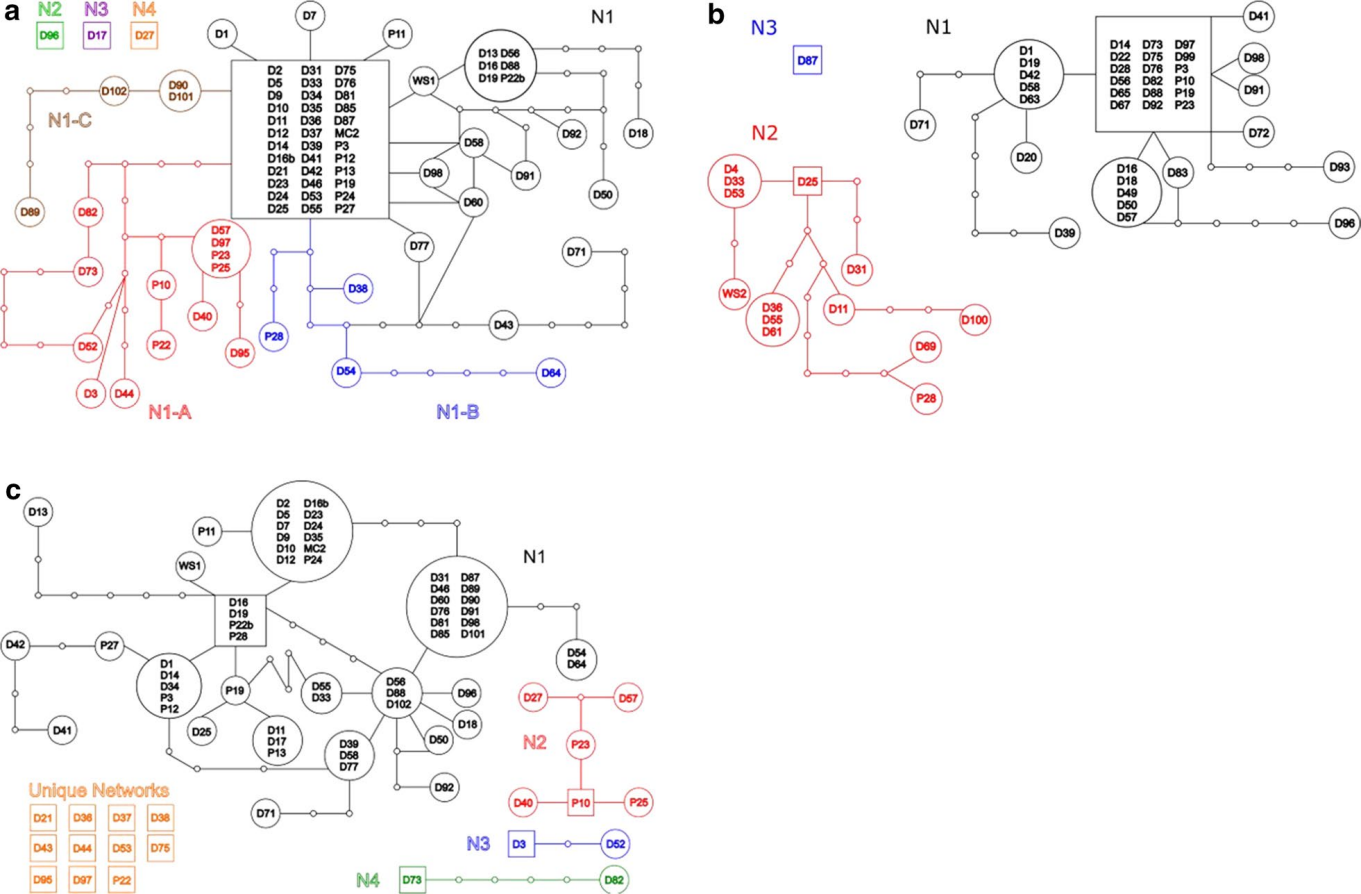
Haplotype networks (N#) of the lichenized green alga *Diplosphaera chodatii* from Payuk Lake, Canada using the internal transcribed spacer regions (ITS) 1, ITS2 and β-actin protein coding gene region markers. Different colors represent the different networks for each of the markers used. Letters followed by numbers correspond to the sample that was collected. Squares represent the first and dominant haplotype designated to a network, circles with samples represent related haplotypes (size is proportional to the number of samples that have the haplotype), and small hollow circles represent single base pair changes from the dominant haplotype

The ITS1 region of the ITS rDNA gene within *Diplosphaera chodatii* contained 36 haplotypes within 80 samples examined (35 haplotypes when excluding the Whiteshell sample, WS1). There were four haplotype networks produced (N1, N2, N3, and N4) based on the total number of base pair changes from the dominant haplotypes computed from TCS (Fig. 1a). Haplotype network 1 (N1) contained 31 haplotypes (30 when excluding the Whiteshell sample), and can be subdivided into three subnetworks within the main network, based on isolated branches that are not connected with other samples within the main network (N1). Subnetwork N1-A contained 10 haplotypes, subnetwork N1-B contained four haplotypes, and subnetwork N1-C contained three haplotypes. Haplotype networks N2, N3, and N4 are considered “unique” networks since they contained only a single haplotype (one sequence) from a single sample.

The ITS2 region of the ITS rDNA gene within *D. chodatii* contained 43 haplotypes within 80 sequences examined (42 haplotypes when excluding the Whiteshell sample, WS1). There were 15 haplotype networks defined (Fig. 1b). There were 21 haplotypes (22 if WS1 is included, 59 sequences) contained in haplotype network 1 (N1), six haplotypes (six sequences) in haplotype network N2, two haplotypes (two sequences) in N3, and two haplotypes (two sequences) in N4. The other 11 haplotype networks were considered “unique” since they contained only a single haplotype from a single sample collected from the lake.

The β-actin gene within *D*. *chodatii* contained 23 haplotypes from 52 sequences examined (22 haplotypes excluding the Whiteshell samples, WS2). There were three haplotype networks defined (Fig. 1c). The first network N1 contained 13 haplotypes (38 sequences), the second network N2 contained 9 haplotypes (13 sequences), and the last network N3 was unique and contained a single haplotype from a single specimen.

### Genetic variation corresponded with the topography of Payuk Lake

**Fig. 2:**
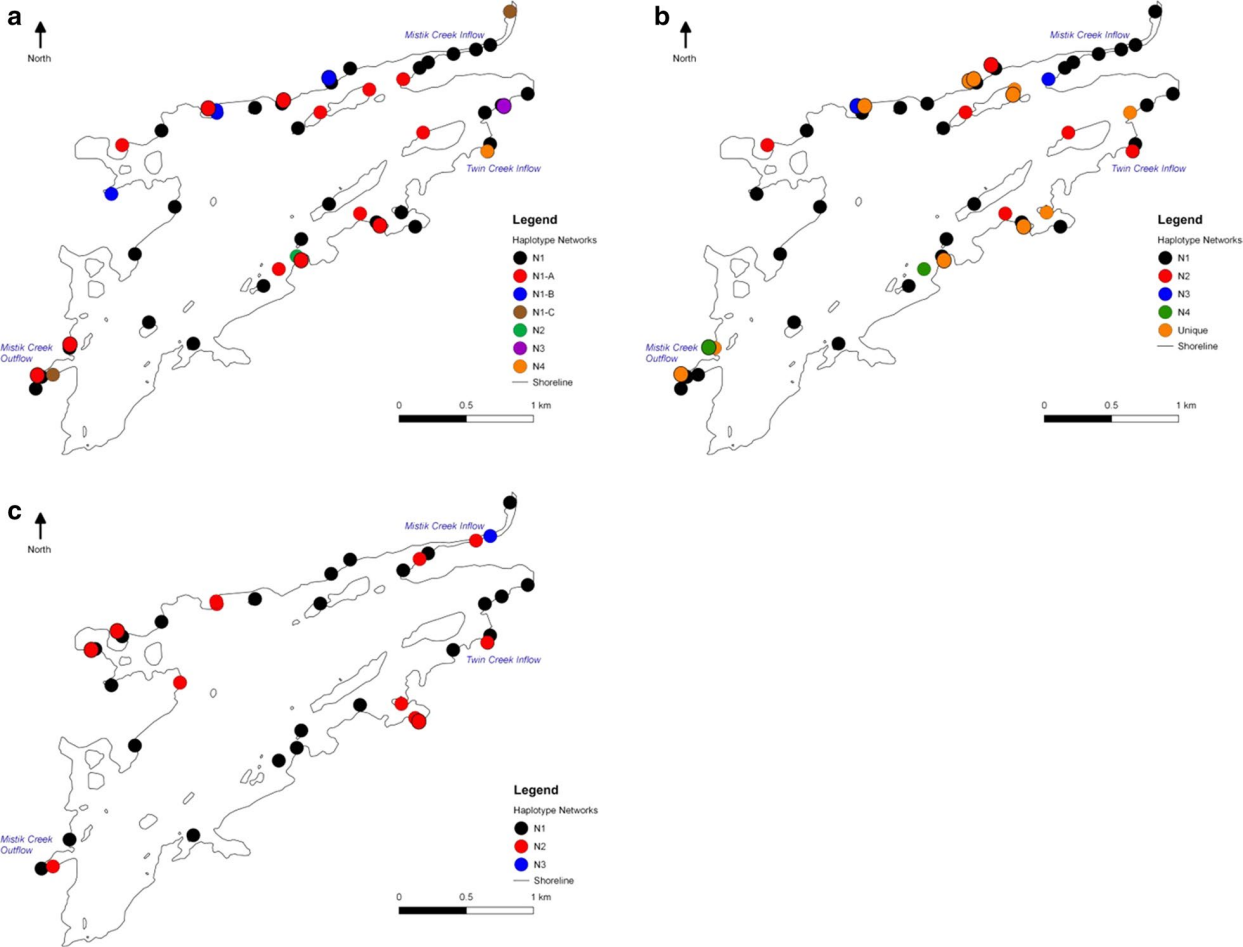
Distribution of haplotype subnetworks of **a** the internal transcribed spacer region 1 (ITS1), **b** the internal transcribed spacer region 2 (ITS2), and **c** β-actin protein gene of lichenized *Diplosphaera chodatii* in Payuk Lake, Manitoba. The colors represent different haplotype networks

Patterns of genetic variation within the ITS1 and ITS2 rDNA and β-actin gene regions corresponded with the topography of Payuk Lake, namely following with the dominant hydrological flow throughout the lake. In the ITS1 rDNA gene region, three unique haplotype networks were found on the Twin Creek (southeast) side of the lake (Fig. 2a). Furthermore, the most dominant haplotype network (N1) was found throughout the lake, in both the inflows (Mistik Creek and Twin Creek), in the middle of the lake, and in the outflow (Mistik Creek). However, even within N1, patterns were seen in the distribution of subnetworks (Fig. 2a). Subnetwork N1-A did not occur in the outflow of the lake and was isolated on islands and secluded points around the lake. Subnetwork N1-B only occurred on the Mistik Creek (northern) side of the lake and in the isolated bay on the northwest side of the lake. Subnetwork N1-C occurred in both the Mistik Creek inflow, as well as the outflow, but not throughout the lake (Fig. 2a).

The ITS2 also had interesting distribution patterns around Payuk Lake. The dominant haplotype network (N1) was found throughout the lake, as well as in both inflows and the outflow (Fig. 2b). Haplotype network 2 (N2) was only within the middle of the lake, on the Twin Creek side and in the Mistik Creek inflow (but not in the creek itself), and not in the outflow. Haplotype network 3 (N3) only occurred on the Mistik Creek (northern) side of the lake and was not present in the outflow. Haplotype network 4 (N4) was found on the Twin Creek (southern) side of the lake and the outflow, but not on the northern shore (Fig. 2b). Unique haplotypes were found throughout the lake. Four unique networks were found in Mistik Creek, four were found within Twin Creek, one was found in the middle region of the lake, and two were found in the outflow.

There were two dominant haplotype networks (N1 and N2) found within the β-actin gene region (Fig. 2c). Both networks were present in Mistik Creek, Twin Creek, the middle regions of the lake, including the isolated bay in the northwest side of the lake, as well as in the outflow. The unique haplotype network N3 was found only in the Mistik Creek inflow, more specifically on a rock within the creek itself.

### Lack of isolation-by-distance may indicate high levels of gene flow and dispersal

Mantel’s test for spatial relatedness indicated that there was no isolation-by-distance occurring among *D. chodatii* samples found within Payuk Lake based on the ITS1, ITS2, and β-actin genes (Table 1), regardless of the geographic distance measure defined (p value > 0.05 in all cases). This large-scale test indicated that gene flow may be occurring in Payuk Lake, but it cannot detect smaller scale variations within the lake. Analysis of Molecular Variance also indicated high gene flow within Payuk Lake for the ITS1, ITS2, and β-actin genes for most landscape hypotheses (*p* value > 0.05), however there was one landscape hypothesis for each gene that showed significant population structure (small scale variation; Table 2). For both the ITS1 and ITS2 genes, the “Wind” landscape hypothesis showed significant variation within populations (PhiPT = 0.031, p-value = 0.04 and PhiPT = 0.016, p-value = 0.05 respectively). In contrast, based on the spatial distribution of the genetic variation in the β-actin gene region, the “Hydrology” landscape hypothesis indicated significant variation within populations (p-value = 0.02, Table 2).

**Table 1:** Summary of Mantel tests for the ITS1 and ITS2 rDNA and Actin genes of *Diplosphaera chodatii*

Gene	Distance measure	Mantel Stat	P-value
Actin	Euclidean	−0.073	0.886
	Shoreline	0.019	0.271
	Network Euclidean	−0.103	0.941
	Network path	−0.115	0.923
ITS1	Euclidean	−0.066	0.861
	Shoreline	0.080	0.102
	Network Euclidean	−0.070	0.865
	Network path	−0.090	0.908
ITS2	Euclidean	−0.090	0.941
	Shoreline	−0.003	0.481
	Network Euclidean	−0.086	0.894
	Network path	−0.061	0.773

**Table 2:** Summary of AMOVA of four different landscape hypotheses

Gene	Population classification	AMOVA analysis	DF	MS	Estimated variation	% Total molecular variation	PhiPT	P-value
Actin	Hydrology	Among Pop.	3	0.783	0.030	7%		
		Within Pop.	47	0.407	0.407	93%	0.069	*0.02*
	Wind	Among Pop.	3	0.425	0.000	0%		
		Within Pop.	47	0.430	0.430	100%	−0.001	0.45
	In–Out-Bay	Among Pop.	2	0.619	0.013	3%		
		Within Pop.	48	0.422	0.422	97%	0.030	0.16
	Bay Topography	Among Pop.	4	0.415	0.000	0%		
		Within Pop.	46	0.431	0.431	100%	−0.004	0.47
ITS1	Hydrology	Among Pop.	3	0.513	0.007	2%		
		Within Pop.	75	0.389	0.389	98%	0.016	0.18
	Wind	Among Pop.	3	0.620	0.012	3%		
		Within Pop.	75	0.385	0.385	97%	0.031	*0.04*
	In–Out-Bay	Among Pop.	2	0.426	0.002	0%		
		Within Pop.	76	0.393	0.393	100%	0.004	0.36
	Bay Topography	Among Pop.	4	0.350	0.000	0%		
		Within Pop.	74	0.397	0.397	100%	−0.008	0.53
ITS2	Hydrology	Among Pop.	3	0.418	0.000	0%		
		Within Pop.	75	0.476	0.476	100%	−0.007	0.64
	Wind	Among Pop.	3	0.614	0.008	2%		
		Within Pop.	75	0.469	0.469	98%	0.016	*0.05*
	In–Out-Bay	Among Pop.	2	0.348	0.000	0%		
		Within Pop.	76	0.478	0.478	100%	−0.014	0.74
	Bay Topography	Among Pop.	4	0.436	0.000	0%		
		Within Pop.	74	0.476	0.476	100%	−0.006	0.58

The genes analysed were the ITS1 and ITS2 regions of the rDNA gene and the β-actin (Actin) gene in lichenized *Diplosphaera chodatii* from Payuk Lake, Manitoba

*DF* degrees of freedom, *MS* mean square error

Significant values are italics

Akaike’s Information Criterion supported the results of the AMOVA (Table 3). The best landscape hypothesis for the ITS1 and ITS2 gene regions was “Wind” (weights = 0.38 for both ITS1 and ITS2). Additionally, for the ITS1 gene region the “In–Out-Bay” (ΔAIC_c_ = 0.49, weight = 0.30) and “Hydrology” (ΔAIC_c_ = 0.87, weight = 0.25) models showed good support (Table 3). The ITS2 gene region showed good support in the “In–Out-Bay” model (ΔAIC_c_ = 0.32, weight = 0.32). The best landscape hypothesis for the β-actin gene region was “Hydrology” (weight = 0.47), with “In–Out-Bay” as the second-best model (ΔAIC_c_ = 0.51, weight = 0.36; Table 3).

**Table 3:** Summary of the corrected Akaike’s Information Criterion (AICc) for landscape hypotheses within Payuk Lake, Manitoba, Canada

Gene	Landscape Hypothesis	Parameters	K	N	AICc	ΔAICc	Weight
Actin	In–Out-Bay	inflow + outflow + mistik + bay	3	51	−40.59	0.51	0.36
	*Hydrology*	*mistik + twin + middle + outflow*	*4*	*51*	*−41.11*	*0.00*	*0.47*
	Wind	mistik + twin + wind + outflow	4	51	−38.33	2.78	0.12
	Bay Topography	mistik + twin + middle + bay + outflow	5	51	−36.84	4.26	0.06
ITS1	In–Out-Bay	inflow + outflow + mistik + bay	3	79	−70.45	0.49	0.30
	Hydrology	mistik + twin + middle + outflow	4	79	−70.07	0.87	0.25
	*Wind*	*mistik + twin + wind + outflow*	*4*	*79*	*−70.94*	*0.00*	*0.38*
	Bay Topography	mistik + twin + middle + bay + outflow	5	79	−67.40	3.54	0.07
ITS2	In–Out-Bay	inflow + outflow + mistik + bay	3	79	−55.13	0.32	0.32
	Hydrology	mistik + twin + middle + outflow	4	79	−54.13	1.32	0.19
	*Wind*	*mistik + twin + wind + outflow*	*4*	*79*	*−55.45*	*0.00*	*0.38*
	Bay Topography	mistik + twin + middle + bay + outflow	5	79	−52.95	2.50	0.11

The genes used were the β-actin protein (Actin) gene and internal transcribed spacer regions (ITS1 and ITS2) of the rDNA gene within lichenized *Diplosphaera chodatii*

*K* number of parameters, *n* sample size, *AIC*_*c*_ corrected AIC value, *ΔAIC*_*c*_ difference in the corrected AIC value when compared to the minimum AIC value, and *Weight* model weights

Significant results are italics

## Discussion

### Conflicting modes of dispersal inferred by ITS rDNA and β-actin

**Fig. 3:**
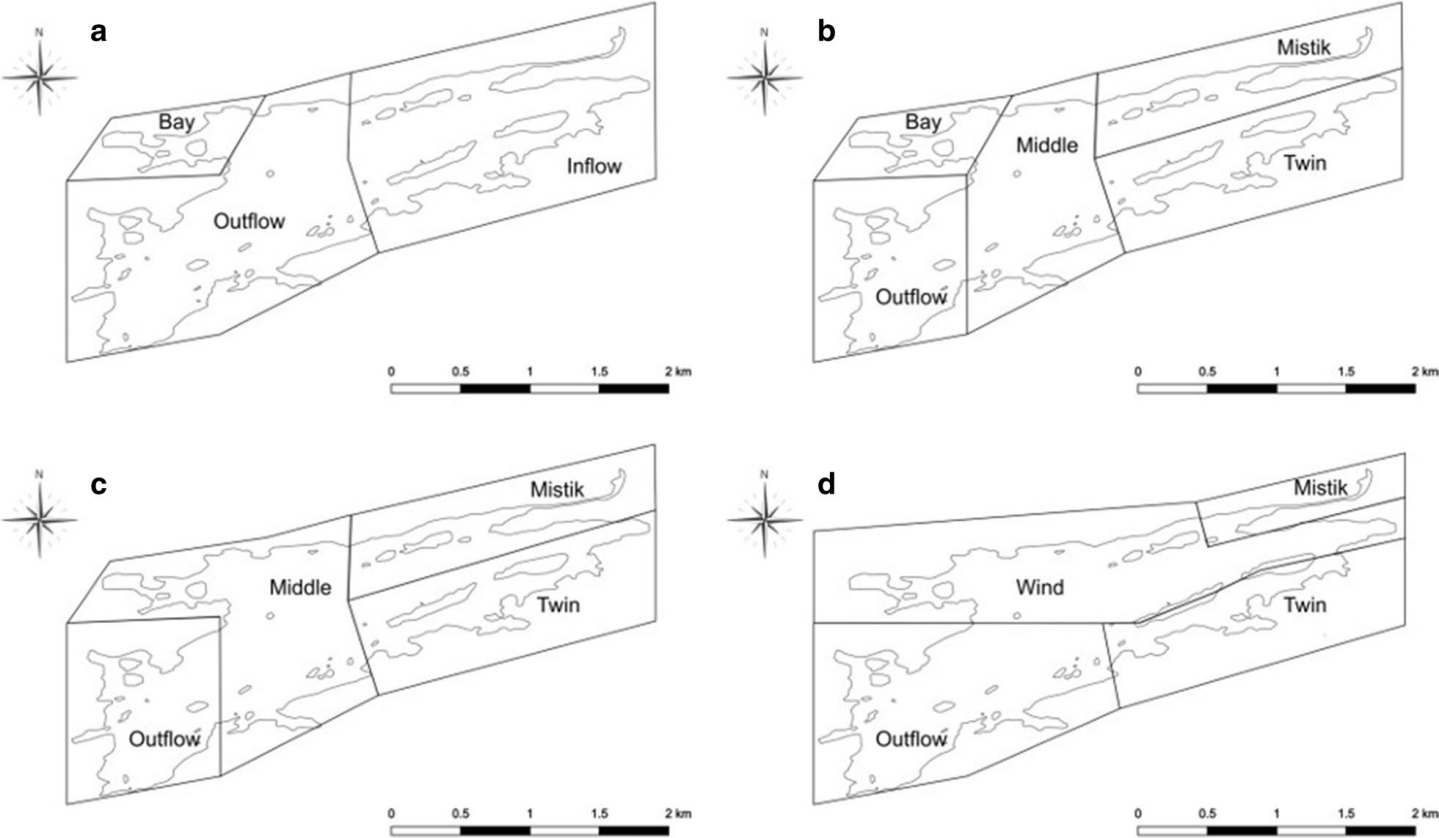
Map of Payuk Lake divided into geographic sections to show the definitions of populations for four ecologically meaningful hypotheses used in analyses of population structure and gene flow. **a** Inflow-outflow-bay; **b** Bay topography; **c** Hydrology; **d** Wind. Base map source: publicly available datasets offered through CanVec (GeoGratis, Natural Resources Canada)

Gene flow was inferred to be occurring in the lichenized *D. chodatii* based on both the ITS (ITS1 and ITS2) rDNA and β-actin gene sequences (Table 2). This was consistent with the finding that there was no support for isolation-by-distance (Table 1). However, although gene flow was inferred, one landscape hypothesis (Wind, Hydrology, In–Out-Bay, and Bay topography) for each gene exhibited significant support based on significant p-values in the AMOVAs (Table 2). The variation between populations for both ITS partitions was consistent with the Wind landscape hypothesis (Fig. 3d). The variation for the β-actin gene was consistent with the Hydrology landscape hypothesis (Fig. 3c). The Wind landscape hypothesis assumed predominant westerly wind patterns across the surface of the lake (and therefore wind dispersal was inferred). The Hydrology landscape hypothesis subdivided the lake based on the net flow of water due to topography (and therefore water dispersal was inferred). Therefore, the significant distribution of β-actin genetic variation is explained by water dispersal while the ITS partitions are explained by wind dispersal. We note that differences in gene variation consistent with these two hypotheses is not definitive evidence for these as the primary dispersal mechanisms. Such inference, based on assumptions about patterns of water and wind flow in the environment, are typical steps in developing understanding in an emergent field such as landscape genetics [42]. Future collaborations with hydrologists and meteorologists to measure and model fine-scale water and wind flow would be necessary to elucidate mechanisms of dispersal of *D. chodatii* more definitively in this system. Nonetheless, parsimony suggests that flows of water and air are likely contributing in some way to distribution of haplotypes within Payuk Lake.

The conflicting modes of dispersal inferred by the spatial distribution of genetic variation and population structure can be explained if the ITS rDNA and β-actin genes are governed by different mutational processes [46]. Even though gene flow is thought to affect all nuclear loci in the same way, the mutation rate of actin genes is more than two times faster than rDNA sequences in fungi and plants, but actin evolves at one-fifth the rate of rDNA sequences in animals [5]. This information likely refers to the coding regions of rDNA rather than the spacer regions. Nevertheless, different rates of evolution may result in some loci showing low levels of population structure while others show high levels of structure [46]. In this study, the ITS2 was found to be the most variable of the gene regions examined due to a high frequency of SNPs and indels. If the β-actin is more conserved than the ITS rDNA regions, perhaps not enough variation existed to be detected within the Wind landscape hypothesis, leading to the difference in significant population structures between the ITS and β-actin haplotype networks. Using more variable DNA markers, such as microsatellites or mtDNA, in tandem with these housekeeping markers may help to reveal more fine-scale variation between the samples, allowing for more population structure to be detected [60]. Additionally, increasing the sampling size (more thalli within each population of each landscape hypothesis), and looking at populations across different lakes within a larger region, may help to resolve more unique haplotypes into more dominant haplotype clusters, or by increasing the frequency of the unique haplotypes resulting in less rare haplotypes. Finally, it is possible that the effects of wind vs. water dispersal are acting on genetic diversity at different spatial extents than measured here [42].

### Population structure was present between the subdivided populations

Significant differences in population structure were present between the Outflow and the other populations in the landscape hypothesis (Mistik Creek, Twin Creek, and Wind) using the ITS1 rDNA. In all population comparisons, there were two shared ITS1 haplotypes, with the rest of the haplotypes present in each population being unique, that is, a single occurrence of the haplotype. The same situation also occurred between significant pairwise population comparisons in the ITS2, and in the β-actin. In all cases, dominant haplotypes were moving throughout the lake (Fig. 2). The significant population structure, despite a low level of gene flow, may be a result of the presence of too many unique haplotypes among all the populations, which may be the result of several factors. If unique haplotypes were not shared between populations, more distinct population structure would be observed even if dominant haplotypes were being shared, differentiating the populations. In the same study region, Robertson and Piercey-Normore [43] also found many unique haplotypes (five of eleven haplotypes) within *Cladonia arbuscula*, suggesting population structure is common within a small spatial extent.

The presence of unique haplotypes may be the result of run-off from the surrounding bedrock cliffs bringing in new haplotypes. Additionally, animal vectors, such as waterfowl and fishes, as well as insects [28] may be introducing new haplotypes from nearby lakes, bogs, or water sources. Kristiansen [28] noted that many freshwater and terrestrial algae are introduced to new habitats via animal or wind vectors. Small animals frequent the rocky shores of Payuk Lake and include loons, ducks, frogs, and otters. Furthermore, human activity, such as boating and fishing, may transport and introduce new haplotypes between lakes and streams. Wind would more likely carry fungal ascospores (rather than thallus lobes) produced from perithecia of *D. luridum*, which may be released while the lake water level is low and the lichen is above water. Ascospores, after germination, would achieve a successful lichenization with *D. chodatii*, either free-living or from surrounding lichen thalli, resulting in a wide variety of haplotypes being preserved in the lake due to the lichenization between fungal ascospores and *D. chodatii*. *Diplosphaera chodatii* was observed to be free-living (see Additional file 1: Figure S1.2 and Table S2). If free-living *D. chodatii* was present on the surface of *Dermatocarpon luridum* thalli, and detected using algal specific markers, it could have overestimated the level of genetic variation observed in lichenised *D. chodatii* around Payuk Lake. Due to the availability of small protected spaces in between thallus lobes, Muggia et al. [35] suggested that lichen thalli could provide protected niches for other photobionts or algal species to inhabitant, so the potential for free-living *D. chodatii* to be present superficially on the thallus is likely.

This is important because clonal dispersal (from fragmentation) would not be enough alone to explain the high genetic variation observed, indicating that immigration of non-local haplotypes must be occurring [50]. Also, land barriers to gene flow, such as islands and rocks or aquatic vegetation, may trap perspective *D. chodatii* in various areas of the lake [58], leading to unique populations. This may be especially prominent in the southwestern bay if water currents bring in algae but back currents do not exist to allow the algae to return to the main hydrological flow. However, unless there was a high biomass of free-living *D. chodatii* nestled within thallus niches, the algal specific markers would most likely pick up the DNA of the most predominant alga present, which is the lichenised photobiont. Thus, the thallus DNA extracted in this study may truly represent that of the photobiont, while free-living *D. chodatii* may not have contributed to much of the DNA extracted and hence the genetic variation observed. Furthermore, having complete bathymetric data, as well as water current speed and depth and wind speed and direction, would allow for complete understanding of geological processes occurring within Payuk Lake to better understand barriers to gene flow and dispersal.

Lastly, spatial scale and sampling may contribute to the high presence of unique haplotypes and spatial variation of *D. chodatii* within the lake. If the spatial extent is too large, complete genetic variation may be underestimated as sampling may not have been sufficient at the population level [53, 42]. Again, increasing sampling size may help to ensure the best representation of the genetic variation in *D. chodatii* at that time is accounted for in order to see true population structure within Payuk Lake.

### High level of genetic variation was present in a small spatial extent

Rarefaction is used to detect the cumulative allelic diversity within a population, where increasing sample sizes beyond a sample size threshold is unlikely to add additional alleles [53]. Werth [53] suggests that the sampling size should be high enough to account for the variation, with about 20 individuals needed per population to detect all of the genetic variation present within the site. In the case of this study, 102 *Dermatocarpon luridum* thalli were sampled within Payuk Lake (treating Payuk Lake as a single “population” according to the definition of Werth [53]); however maximum genetic diversity (a plateau in the rarefaction curve) was not obtained in any of the ITS markers (ITS1 and ITS2) nor the β-actin. Thus, even though 20 samples were used as recommended by Werth [53], the sample size of this study may not have been high enough based on the spatial extent to account for most of the variation. The insufficient sampling may account for the large number of unique haplotypes resulting in an interpretation of high population structure. If more sampling were conducted, more gene flow may have been inferred. The spatial scale and method of haplotype inference may also affect the cross comparison of results. Robertson and Piercey-Normore [43] found five unique fungal haplotypes and six common haplotypes in a small spatial extent (2 km) while Lindblom and Ekman [30] found ten haplotypes within 3 km. Payuk Lake is approximately 4 km long, and 2 km wide [34]. However, Robertson and Piercey-Normore [43] used the pattern and presence/absence of introns in the fungal 18S rDNA of *Cladonia arbuscula* but Lindblom and Ekman [30] used the DNA sequenced from the ITS and IGS regions of the ribosomal DNA. The finer detail in the variation of the spacer regions would reveal more genetic diversity than using presence/absence alone.

The level of genetic variation can also be confounded by the nature of the organism. *Diplosphaera chodatii* is a haploid organism, which would result in having a single set of chromosomes (and therefore a single set of alleles within that site in the DNA) present within the DNA, thus requiring twice as intensive sampling as would be required for diploid organisms [53]. Furthermore, gene regions such as the ITS, which occur as tandem repeat regions within the DNA, may have paralogs due to the absence and presence of introns [45], resulting in an increased allelic variation and higher frequency of haplotypes. One way to alleviate this would be to use more variable, multi-locus markers, such as microsatellites [53] that result in multiple banding patterns [60]. Microsatellite markers, or simple sequence repeats (SSR), can identify more complex patterns in haploid genomes than single locus genetic markers such as the ITS or β-actin, and could also address the homogenization of gene flow from different mutation rates as suggested by Slatkin [46].

Sequencing success can also affect the final sample size of sequences obtained from specimens collected. Although 102 lichen thalli were collected, only 79 thalli were successfully sequenced using the ITS rDNA (ITS1 and ITS2; 77% success), and 51 thalli were successfully sequenced using the β-actin gene marker (50% success), thus resulting in a smaller effective sample size. Difficulty in sequencing these gene regions may be due to sequencing miscalls or errors introduced in PCR amplification [14] or from the presence of multiple strains within a thallus [38]. The steps needed to successfully sequence DNA can damage the sample [8], especially during the sample preparation (PCR, purification, and cycle-sequencing). Noise in the phylogenetic signal of the DNA sequence alignment could also cause discrepancies in effective sample sizes. Many nucleotide substitutions can cause the apparent distances between sequences to be greater than the actual genetic distances, resulting in apparent saturation [39]. This would give the indication that there are many more samples than there are actually present due to low resolution of variants.

## Conclusions

We inferred that the mode of dispersal for the semi-aquatic lichen *Dermatocarpon luridum* could have been either wind and/or water due to the conflicting interpretations of our landscape hypotheses based on ITS rDNA and β-actin DNA sequences of the photobiont markers. If more sampling occurred to overcome the limitation of the study, we may have found that more population structure was present due to fewer unique haplotypes supporting water vectored dispersal. Additional sampling and sequencing of more variable markers and using the ITS rDNA and β-actin markers in combination, as well as the use of SSR markers, may help to alleviate these discrepancies. While landscape genetics holds promise for inferring ecological processes, we must be mindful of the limitations of inference [42] and the limited number of studies to date that have applied landscape genetics to lichens [29].

While both landscape and wind hypotheses for dispersal were supported by different genes, population structure within the photobiont was present between the subdivided populations in the lake. While there are limitations to this study, this is the first study to use spatial modelling to infer dispersal of the photobiont in semi-aquatic lichens within an aquatic environment and serves as a proof of concept for the method. Previous lichen landscape genetic studies have focused on the fungal component of epiphytes (e.g., [48, 54, 55]) and rock-dwelling lichens at high altitudes (e.g., [7, 24]). The use of spatial modelling may help to gain a better understanding of methods of lichen dispersal and gene flow within a habitat where a lichen relies on both lake water and atmospheric dehydration for survival. The photobiont in this lake system has been studied previously between continents [20], providing a unique opportunity now to compare across spatial scales. This study provided the first extensive study of the genetic variability of lichenized *Diplosphaera chodatii* on a single lake using local spatial scales to better understand gene flow and potential dispersal of *D. luridum*.

Despite lack of clear inference of one dispersal mechanism over another, our study shows that genetic diversity of a single algal species, associated with a semi-aquatic lichen, is quite high within a small spatial extent. Differences in photobiont haplotypes throughout the lake may also identify unique populations of *D. luridum* within the lake, which would have implications for conservation around the lake and raise questions about the possibility of free-living *D. chodatii* contributing to the lichenized population. Other organisms such as invertebrates, aquatic plants, and other lichens, including the endangered *Leptogium rivulare* (COSEWIC [10]) and *Peltigera hydrothyria* (COSEWIC [11]), may be studied to incorporate a broader context for conservation as they both require aquatic environments for survival and dispersal.

## Materials and methods

### Field collections

This study took place in Payuk Lake, which is a boreal lake in northern Manitoba, surrounded by igneous substrata, which is fed by two boreal creeks: Mistik Creek and Twin Creek [34]. The Mistik Creek watershed is located approximately 32 km southeast of Flin Flon, Manitoba, Canada. The collection of 102 lichen thallus (and therefore photobiont) samples was made in 34 collection sites from Payuk Lake, Manitoba (Additional file 1: Figure S1.1, Appendix S1 in Additional file 1), with three replicates collected within three meters of each other at each site. These numbers resulted in a photobiont sample size high enough to compare the genetic diversity in a local population study [53]. Thallus collections were made in the inflow of Payuk Lake at Mistik Creek (18 samples at 6 sites), the inflow of Payuk Lake at Twin Creek (6 samples at 2 sites), Payuk Lake (72 samples at 24 sites), the outflow of Payuk Lake into Mistik Creek (6 samples at 2 sites). Three additional samples were collected from the Whiteshell Provincial Park (approximately 882 km southeast of Payuk Lake) in July 2015 (Permit number PP-PHQ-15-015), as a reference from a geographically separated population from that of Payuk Lake. The 34 collection sites were recorded using a Garmin GPS map 76C x GPS unit (datum NAD83, UTM zone 14 N) and were at least 25 m apart. They were selected based on accessibility and the presence of *Dermatocarpon luridum* thalli along the lake margin. Portions of each *D*. *luridum* thallus were moistened with lake water to scrape the thallus from the rock with a knife without breaking it into many pieces and placed into separate ziplock bags. Lichen samples were cleaned of debris and 1 cm thallus portions (approximately < 20 mg dry weight) were placed in 1.5 mL Eppendorf tubes and stored at −20 °C for use in molecular analyses, while the rest of the thallus was transferred to paper envelopes to be deposited as vouchers in the cryptogamic division of the University of Manitoba Herbarium (WIN).

To determine whether free-living *D. chodatii* may be present within our study environment, the surface of the rock, which was colonized by the lichen thalli, was scraped with a clean toothbrush and rinsed into a vial containing 20 mL of sterile MilliQ water. The samples were then stored at −20 °C for further microscopic examination and culturing.

### DNA sequencing

The thallus samples of *D*. *luridum* designated for molecular processing (approximately < 20 mg dry weight) were crushed to a powder in liquid nitrogen. DNA extraction was performed using DNeasy Plant Mini Kit (Qiagen Inc., Toronto, Ontario), following the manufacturer’s protocol. Polymerase chain reaction (PCR) was performed to amplify two algal loci (Table 4): the algal internal transcribed spacer ribosomal DNA (ITS rDNA) region following that of Fontaine et al. [19], as well as the internal primers Dch-5.8S-F, Dch-5.8S-R, Dch-ITS-R; and algal β-actin type 1 gene (Actin) using the forward and reverse primers Dch-βactin-F and Dch-βactin-R. The ITS gene region was amplified in 20 μL (for checking amplification success) and 50 μL (for sequencing preparation) reactions using 1X PCR Buffer (200 mM Tris–HCl, pH 8.4, 500 mM KCl, 2 mM MgCl_2_; GeneDireX, FroggaBio, Toronto, Ontario, Canada), 2 Units Taq DNA Polymerase (GeneDireX, FroggaBio, Toronto, Ontario, Canada), 200 μM of each dNTP (GeneDireX, FroggaBio, Toronto, Ontario, Canada), 0.5 μM of each of the forward and reverse primers, and 5–25 ng of DNA template. The PCR was performed on a Biometra T-Gradient thermal cycler (Montreal Biotech Inc., Dorval, Quebec, Canada) using an initial 3 min at 94 °C, followed by 30 cycles of denaturation at 94 °C for 1 min, annealing at 58.5 °C for 30 secs, and elongation at 72 °C for 45 secs, and ending with a hold at 6 °C.

**Table 4:** Primers used for polymerase chain reaction (PCR) and DNA sequencing

Primer Name	Sequence	Gene	Purpose	Source
STICHO-ITS-F-5′	5′-GGATCATTGAATCTATCAACAAC-3′	ITS rDNA	PCR and Sequencing	[19]
Dch-ITS-R	5′-TGGTGGCCGAGCGGACGATT-3′	ITS rDNA	PCR and Sequencing	This study
ITS4-3′	5′-TCCTCCGCTTATTGATATGC-3′	ITS rDNA	PCR and Sequencing	[59]
Dch-5.8S-F-5′	5′-CGGATATCTTGGCTCCCGCAT-3′	5.8S Ribosomal region in ITS	Sequencing	This study
Dch-5.8S-R-3′	5′-ACCGAAGTCTCGAGCGCAATA-3′	5.8S Ribosomal region in ITS	Sequencing	This study
Dch-βactin-F	5′-CAACACAGCGAGTGCCCTAT-3′	β-actin protein	PCR and Sequencing	This study
Dch-βactin-R	5′-TGTACCGCACCTGTAAGCGC-3′	β-actin protein	PCR and Sequencing	This study

The genes used were the Internal transcribed spacer (ITS) regions of rDNA and β-actin protein genes

The actin gene region was amplified in 20 μL and 50 μL reactions using 1X PCR Buffer (200 mM Tris–HCl, pH 8.4, 500 mM KCl, 2 mM MgCl_2_; GeneDireX, FroggaBio, Toronto, Ontario, Canada), 2.5 Units Taq DNA Polymerase (GeneDireX, FroggaBio, Toronto, Ontario, Canada), 200 μM of each dNTP (GeneDireX, FroggaBio, Toronto, Ontario, Canada), 0.5 μM of each of the forward and reverse primers, and 5–25 ng of DNA template. The PCR was performed on a Biometra T-Gradient thermal cycler (Montreal Biotech Inc, Dorval, Quebec, Canada) using an initial 3 min at 94 °C, followed by a touchdown sequence of 2 cycles of denaturation at 94 °C for 1 min, annealing at 54 °C for 30 secs, and elongation at 72 °C for 30 secs; 2 cycles of denaturation at 94 °C for 1 min, annealing at 53 °C for 30 secs, and elongation at 72 °C for 30 secs; then 26 cycles of denaturation at 94 °C for 1 min, annealing at 52 °C for 30 secs, and elongation at 72 °C for 30 secs; and ending with a hold at 6 °C.

The PCR product was prepared for DNA sequencing by ethanol precipitation, cleaned by excising the DNA bands from gel electrophoresis on a 1% agarose gel in 1X Tris–borate (TBE) buffer, and purified using the Wizard SV-Gel and PCR Clean-up kit (Promega, Madison, Wisconsin, USA) following manufacturer’s directions. Resuspension of the cleaned PCR product was performed in 32 μL of autoclaved sterile distilled water instead of 50 μL as recommended by the manufacturer. DNA sequencing was performed initially using a cycle-sequence reaction in Big Dye v.3.1 (Applied Biosystems, Foster City, CA) and cleaned using ethylenediaminetetraacetic acid (EDTA), following the manufacturer’s instructions. The product was resuspended in Hi-Di formamide (Applied Biosystems, Foster City, CA) and sequenced using a 3130 Genetic Analyzer (Applied Biosystems, Foster City, CA). The sequences were manually cleaned in ChromasPro v2 (Technelysium Pty Ltd, South Brisbane, Australia) and aligned in MEGA 6 [49]. The sequence data were submitted to GenBank, adding an additional 80 ITS rDNA and 52 β-actin sequences of *Diplosphaera chodatii* to their database (Additional file 1: Appendix S1).

### Algal culturing

To determine whether *D. chodatii* could occur free-living, algae from the rock scrapings were isolated using the Percoll method [23], with modifications. Briefly, rock scrapings was resuspended in 200 μL sterile isotonic buffer (0.3 M sorbitol in 50 mM HEPES, pH 7.5), which was then loaded onto 1.5 mL of 80% Percoll in isotonic buffer in a 10 mL centrifuge tube and spun in a Sorvall Legend X1R centrifuge (Thermo Fisher Scientific, Waltham, MA, USA) at 5000 rpm for 20 min. A total of 400 μL of the green layer (just below the surface of the supernatant) was aliquoted into a new 10 mL centrifuge tube and diluted two-fold in sterile distilled water and centrifuged at 2000 rpm for 10 min. The supernatant was discarded, and the pellet was resuspended in 2 mL of sterile distilled water and 1 drop of Tween 20. The samples were subjected to sonification using a Fisher Vortex Genie 2 (Thermo Fisher Scientific, Waltham, MA, USA) five times. Each sonification round consisted of 1 min steady sonification at 40 kHz, followed by centrifugation at 5000 rpm for 5 min. Finally, the samples were resuspended in 1 mL of sterile distilled water in a 1.5 mL Eppendorf tube.

The isolated algal samples were directly inoculated into flasks containing sterile liquid BBM media [6] and incubated at 20 °C under 24 h light conditions. The algal cultures were examined after 10 months using a Zeiss AxioImager.Z1 Microscope (Carl Zeiss Microscopy, LLC, Thornwood, NY, USA) under bright field (BF) and differential interference contrast (DIC). Photographs of each algal specimen were taken with a Zeiss AxioCam 105 Colour Camera (Carl Zeiss Microscopy, LLC, Thornwood, NY, USA). Algal specimens were identified to genus using Wehr and Sheath [52]. The DNA of the algal cultures was extracted using DNeasy Plant Mini Kit (Qiagen Inc., Toronto, Ontario), following the manufacturer’s protocol and PCR using *Diplosphaera*-specific ITS primers (Table 4) was performed. The PCR product was run using gel electrophoresis on a 1% agarose gel in 1X Tris–borate (TBE) buffer to determine the presence of the ITS band.

### GIS analyses

Baseline GIS data of Payuk Lake were downloaded from the publicly available datasets offered through CanVec (GeoGratis, Natural Resources Canada; https://www.nrcan.gc.ca/earth-sciences/geography/topographic-information/free-data-geogratis/11042, accessed April 2015). A digital elevation model (DEM) of Payuk Lake and surrounding areas was provided by Garry Lux from the Manitoba Government in Flin Flon, Manitoba. All of the data were defined to be in the NAD83 UTM 14 N projection.

To account for movement within the watershed over the landscape, four pairwise measures of distance were defined and calculated between the collected samples: the Euclidean distances within Payuk Lake; the distance along the shoreline of the lake to account for topography; Euclidean distances along a net flow accumulation network; and a path distance along a net flow accumulation network (taking into account water flow direction). Mantel’s test for spatial autocorrelation was performed using these distances. The Euclidean straight-line distance between samples was calculated using the “distance matrix” tool in QGIS 2.18.2 Las Palmas [41]. The distance between sample sites along the shoreline takes into account the curvature of the bays within the lake (as in the perimeter of the lake) and was obtained by converting the lake feature from a polygon to a polyline feature. The locations of the samples were snapped to the shoreline polyline feature. Shortest path analysis was performed using the “v.net.allpairs” command within the QGIS GRASS 7 algorithms in the toolbox to obtain all of the shortest pairwise distances between all samples following the shoreline.

Since water flows along the path of least resistance (i.e., downhill or through a watershed), the distance between all of the samples along this flow accumulation “network” was determined using the “Hydrology” toolset within the “Spatial Analyst” toolbox in ArcGIS 10.2 [13]. Using the DEM of Payuk Lake, a flow direction layer of water through the lake was created using the “Flow Direction” tool. Sinks were identified using the “sinks” tool and removed using the “fill” tool. The flow direction was recalculated once the sinks were removed. Next, a flow accumulation network was created using the “flow accumulation” tool. As water moves within a system, net accumulation of water occurs in the direction of flow within the system. Downstream has a higher net accumulation than upstream due to runoff and stream flow. To create the stream network used in the calculation of distance in the network, flow accumulation was converted to a stream order raster using the “stream order” tool and transformed into a feature class using the “stream to feature” tool. Values of flow accumulation were than extracted from the accumulation raster and added to the stream order feature using the “stream link” tool. The flow lengths (of the network) upstream and downstream were calculated using the “flow length” tool, with the upstream and downstream parameter selected respectively. Raster values (indicating the lengths) were extracted using the “extract values to points” tool within the “Extraction” toolset and exported into Excel. Using the baseline data of Payuk Lake, Mistik and Twin creeks polylines were merged together into a single polyline using the Editor tool extension and added to the flow accumulation polyline feature. The sample locations were then snapped to the closest spot along the new stream polyline using the “snap” tool within the “editing tools” toolset. The raster values of the flow accumulation underneath the samples along the network were extracted (the higher the number, the further downstream the sample was located) using the tool “extract values by points” within the “extraction” toolset. Next, the net flow accumulation polyline feature was imported into QGIS 2.18.2, where the Euclidean distance of the samples along the net flow accumulation network was calculated using the “distance matrix” tool within the “analysis” toolset of vector analysis, using a linear matrix. Lastly, a path distance (equivalent to shoreline distance) was calculated between all of the samples along the flow accumulation network using the same steps to obtain the shoreline distance.

### Data analyses

To ensure pairwise comparison of base pairs for haplotype analyses with all samples (those sequenced in this study and acquired through GenBank), the sequences were trimmed to be all the same length. To determine the relationship of the 102 *Diplosphaera chodatii* samples with each other, along with previous known samples collected from Payuk Lake, maximum likelihood (ML) tests of the ITS rDNA and β-actin alignments were determined in MEGA 6 [49], using all sites with a Kimura 2-parameter model and 1000 bootstrap permutations. Due to the lack of resolution in the ITS rDNA ML results, the ITS rDNA alignment was split into the ITS1 region with partial 5.8S ribosomal DNA gene (1–315 bp) and the ITS2 region with partial 5.8S ribosomal DNA gene (316–607 bp). Pairwise genetic distances for use in Mantel’s test were computed for each of the ITS1, ITS2, and β-actin in MEGA 6 using the Kimura 2-parameter model, 1000 bootstrap permutations, complete deletion of missing base pairs, and using transversions and transitions.

To assess the relatedness of each sequence with each other (i.e., genetic distance), haplotype networks were constructed using TCS 1.21 [9], using the 5th state criterion and 95% probability of parsimony [20]. Each single nucleotide change (base pair change, insertion, or deletion) from the first assessed sequence was considered to be a new haplotype and so on. The haplotype network of each of the ITS1, ITS2, and β-actin genes were then mapped in QGIS to assess the distribution of genetic variation around Payuk Lake.

Populations within Payuk Lake were defined based on ecological and geological hypotheses according to net flow of water through the lake (Fig. 3): the first hypothesis (Inflow-Outflow-Bay; Fig. 3a) subdivides the lake according to an inflow region, and outflow region, and an isolated bay region; the second hypothesis (Bay Topography; Fig. 3b) subdivides the lake based on topographical features, with Mistik Creek and Twin Creek as two separate inflow regions, a middle “mixing” region, an isolated bay, and the Mistik Creek outflow; the third hypothesis (Hydrology; Fig. 3c) subdivides the lake based on the flow of water (which may affect dispersal if lichenized *D*. *chodatii* is dispersed by water), with two inflow regions (Mistik and Twin Creeks), and middle “mixing” region, and the Mistik Creek outflow; and the last hypothesis (Wind; Fig. 3d), adds an element of predominant wind patterns across the surface of the lake with two inflow regions (Mistik and Twin Creek), a wind region which may push surface water against the direction of flow within the middle of the lake (and disperse lichenized *D*. *chodatii* if it is wind dispersed), and the Mistik Creek outflow region. To determine if there is population structure and gene flow of lichenized *D*. *chodatii* within Payuk Lake, AMOVA [15] was performed using GenAlEx v6.501 [36, 37], using 999 permutations, and to obtain a PhiPT fixation index to assess gene flow. Since only one lake was being assessed, the number of populations was determined by how Payuk Lake was subdivided (Fig. 3), and the number of regions was set to one. Mantel’s test was also performed to assess gene flow by means of isolation by distance [33] using the genetic distance and geographical distance matrices obtained earlier. Mantel’s test was performed in R using the “mantel” function from the “ecodist” package, and 999 Monte Carlo permutations.

Lastly, to determine which definition of “population” (and therefore to infer mode of dispersal of the lichen fragments) for *D*. *chodatii* around Payuk Lake best explained the genetic variation and gene flow within the lake, a modified Akaike’s Information Criterion (AIC; [2]) was performed using the sum of squares within populations (output from AMOVA) rather than the error sum of squares from a typical analysis of variance. The corrected AIC (AICc) was used to assess the best definition since the sample size divided by the number of “model” parameters (number of subdivisions within the lake) was less than 40. Levels of significance for all statistical tests were assessed at α < 0.05.

## Supplementary information

**Additional file 1:** Figures and Tables containing collection information, GenBank accession numbers and cultured algal characteristics.

## Data Availability

The sequence data were submitted to GenBank (https://www.ncbi.nlm.nih.gov/nuccore/) and accession numbers are referenced in Additional file 1. All other data generated or analysed during this study are included in this published article [and its Additional files].
