# Supplementary material for: How do genes flow? Identifying potential dispersal mode for the semi-aquatic lichen Dermatocarpon luridum using spatial modelling and photobiont markers
Source: BMC Ecol. 2020 Oct 15;20:56. doi: 10.1186/s12898-020-00324-4 (PMC7565318; doi:10.1186/s12898-020-00324-4)
Supplement: Supplementary file 1 — Additional file 1: Figures and Tables containing collection information, GenBank accession numbers and cultured algal characteristics. [file 12898_2020_324_MOESM1_ESM.docx]

**Title: How do genes flow? Identifying potential dispersal mode for the semi-aquatic lichen *Dermatocarpon luridum* using spatial modelling and photobiont markers**

**Running Title: Inferences about potential dispersal mode for *Dermatocarpon luridum***

**Authors:** Jennifer A. Doering^1^, Tom Booth^1^, Yolanda F. Wiersma^2^, and Michele D. Piercey-Normore^1*^

**Author Affiliations:**

^1^Department of Biological Sciences, University of Manitoba, Winnipeg, MB, Canada R3T 2N2

^2^Department of Biology, Memorial University of Newfoundland, St. John’s, NL, Canada, A1B 3X9

^*^Current: School of Science and Environment, Grenfell Campus, Memorial University of Newfoundland, Corner Brook, NL, A2H 5G4

Corresponding Author: Jennifer A. Doering, Jennifer.Doering@umanitoba.ca, ORCID: 0000-0002-3181-6518, phone: 1-204-930-3456

Appendix S1. Collection sites, samples, and sequence accession numbers used in this study.

|  | Latitude (Decimal degrees) | Longitude (Decimal degrees) |  | GenBank Accession Number | |
| --- | --- | --- | --- | --- | --- |
| Collection Site |  |  | Sample | ITS | Actin |
| Whiteshell | 49.8095 | -95.2384 | WS1 | MF124964 | - |
| Whiteshell | 49.8095 | -95.2384 | WS2 | - | MF124892 |
| Whiteshell | 49.8095 | -95.2384 | WS3 | - | - |
| 1 | 54.6502 | -101.5122 | D1 | MF124939 | MF124907 |
| 1 | 54.6502 | -101.5122 | D2 | MF124949 |  |
| 1 | 54.6502 | -101.5122 | D3 | MF124954 | - |
| 2 | 54.6510 | -101.5104 | D4 | - | MF124896 |
| 2 | 54.6510 | -101.5104 | D5 | MF124961 | - |
| 2 | 54.6510 | -101.5104 | D6 | - | - |
| 3 | 54.6520 | -101.5065 | D7 | MF124962 | - |
| 3 | 54.6520 | -101.5065 | D8 | - | - |
| 3 | 54.6520 | -101.5065 | D9 | MF124963 | - |
| 4 | 54.6524 | -101.5040 | D10 | MF124940 | - |
| 4 | 54.6524 | -101.5040 | D11 | MF124941 | MF124893 |
| 4 | 54.6524 | -101.5040 | D12 | MF124942 | - |
| 5 | 54.6495 | -101.4978 | D13 | MF124943 | - |
| 5 | 54.6495 | -101.4978 | D14 | MF124944 | MF124906 |
| 5 | 54.6495 | -101.4978 | D15 | - | - |
| 6 | 54.6487 | -101.5008 | D16 | MF124945 | MF124912 |
| 6 | 54.6487 | -101.5008 | D16b | MF124946 | - |
| 6 | 54.6487 | -101.5008 | D17 | MF124947 | - |
| 6 | 54.6487 | -101.5008 | D18 | MF124972 | MF124905 |
| 7 | 54.6482 | -101.5027 | D19 | MF124948 | MF124910 |
| 7 | 54.6482 | -101.5027 | D20 | - | MF124909 |
| 7 | 54.6482 | -101.5027 | D21 | MF124950 | - |
| 8 | 54.6461 | -101.5020 | D22 | - | MF124908 |
| 8 | 54.6461 | -101.5020 | D23 | MF124951 | - |
| 8 | 54.6461 | -101.5020 | D24 | MF124952 | - |
| 9 | 54.6456 | -101.5022 | D25 | MF124953 | MF124894 |
| 9 | 54.6456 | -101.5022 | D26 | - | - |
| 9 | 54.6456 | -101.5022 | D27 | MF125004 | - |

Samples D (*Dermatocarpon luridum* from Payuk Lake) and WS (*D. luridum* from Whiteshell) were collected and sequenced in this study and samples with P (from Payuk Lake) and MC (Mistik Creek) were collected from Fontaine et al. (2013). GenBank Accession Numbers are indicated and a dash refers to samples which were not sequenced. Collection site numbers correspond with those in Figure 1.

Appendix S1 continued.

|  | Latitude (Decimal degrees) | Longitude (Decimal degrees) |  | GenBank Accession Number | |
| --- | --- | --- | --- | --- | --- |
| Collection Site |  |  | Sample | ITS | Actin |
| 10 | 54.6450 | -101.5061 | D28 | - | MF124937 |
| 10 | 54.6450 | -101.5061 | D29 | - | - |
| 10 | 54.6450 | -101.5061 | D30 | - | - |
| 11 | 54.6404 | -101.5103 | D31 | MF124999 | MF124903 |
| 11 | 54.6404 | -101.5103 | D32 | - | - |
| 11 | 54.6404 | -101.5103 | D33 | MF124998 | MF124895 |
| 12 | 54.6413 | -101.5119 | D34 | MF124955 | - |
| 12 | 54.6413 | -101.5119 | D35 | MF124956 | - |
| 12 | 54.6413 | -101.5119 | D36 | MF124957 | MF124897 |
| 13 | 54.6498 | -101.5205 | D37 | MF124967 | - |
| 13 | 54.6498 | -101.5205 | D38 | MF124966 | - |
| 13 | 54.6498 | -101.5205 | D39 | MF125003 | MF124911 |
| 14 | 54.6508 | -101.5184 | D40 | MF124958 | - |
| 14 | 54.6508 | -101.5184 | D41 | MF124959 | MF124933 |
| 14 | 54.6508 | -101.5184 | D42 | MF124960 | MF124931 |
| 15 | 54.6494 | -101.5161 | D43 | MF124970 | - |
| 15 | 54.6494 | -101.5161 | D44 | MF124969 | - |
| 15 | 54.6494 | -101.5161 | D45 | - | - |
| 16 | 54.6483 | -101.5261 | D46 | MF124974 | - |
| 16 | 54.6483 | -101.5261 | D47 | - | - |
| 16 | 54.6483 | -101.5261 | D48 | - | - |
| 17 | 54.6479 | -101.5292 | D49 | - | MF124914 |
| 17 | 54.6479 | -101.5292 | D50 | MF124976 | MF124913 |
| 17 | 54.6479 | -101.5292 | D51 | - | - |
| 18 | 54.6477 | -101.5337 | D52 | MF124975 | - |
| 18 | 54.6477 | -101.5337 | D53 | MF124968 | MF124899 |
| 18 | 54.6477 | -101.5337 | D54 | MF124977 | - |
| 19 | 54.6451 | -101.5444 | D55 | MF124979 | MF124898 |
| 19 | 54.6451 | -101.5444 | D56 | MF124978 | MF124916 |
| 19 | 54.6451 | -101.5444 | D57 | MF124981 | MF124919 |
| 20 | 54.6462 | -101.5399 | D58 | MF124980 | MF124915 |
| 20 | 54.6462 | -101.5399 | D59 | - | - |
| 20 | 54.6462 | -101.5399 | D60 | MF124982 | - |
| 21 | 54.6442 | -101.5474 | D61 | - | MF124900 |
| 21 | 54.6442 | -101.5474 | D62 | - | - |
| 21 | 54.6442 | -101.5474 | D63 | - | MF124918 |

Appendix S1 continued.

|  | Latitude (Decimal degrees) | Longitude (Decimal degrees) |  | GenBank Accession Number | |
| --- | --- | --- | --- | --- | --- |
| Collection Site |  |  | Sample | ITS | Actin |
| 22 | 54.6418 | -101.5454 | D64 | MF1249783 | - |
| 22 | 54.6418 | -101.5454 | D65 | - | MF124917 |
| 22 | 54.6418 | -101.5454 | D66 | - | - |
| 23 | 54.6422 | -101.5375 | D67 | - | MF124924 |
| 23 | 54.6422 | -101.5375 | D68 | - | - |
| 23 | 54.6422 | -101.5375 | D69 | - | MF124901 |
| 24 | 54.6379 | -101.5425 | D70 | - | - |
| 24 | 54.6379 | -101.5425 | D71 | MF124984 | MF124922 |
| 24 | 54.6379 | -101.5425 | D72 | - | MF124921 |
| 25 | 54.6314 | -101.5496 | D73 | MF124985 | MF124920 |
| 25 | 54.6314 | -101.5496 | D74 | - | - |
| 25 | 54.6314 | -101.5496 | D75 | MF124965 | MF124923 |
| 26 | 54.6320 | -101.5354 | D76 | MF124987 | MF124935 |
| 26 | 54.6320 | -101.5354 | D77 | MF124986 | - |
| 26 | 54.6320 | -101.5354 | D78 | - | - |
| 27 | 54.6360 | -101.5275 | D79 | - | - |
| 27 | 54.6360 | -101.5275 | D80 | - | - |
| 27 | 54.6360 | -101.5275 | D81 | MF124989 | - |
| 28 | 54.6372 | -101.5258 | D82 | MF124988 | MF124926 |
| 28 | 54.6372 | -101.5258 | D83 | - | MF124925 |
| 28 | 54.6372 | -101.5258 | D84 | - | - |
| 29 | 54.6527 | -101.5023 | D85 | MF124994 | - |
| 29 | 54.6527 | -101.5023 | D86 | - | - |
| 29 | 54.6527 | -101.5023 | D87 | MF124993 | MF124936 |
| 30 | 54.6550 | -101.5002 | D88 | MF124992 | MF124927 |
| 30 | 54.6550 | -101.5002 | D89 | MF124991 | - |
| 30 | 54.6550 | -101.5002 | D90 | MF124990 | - |
| 31 | 54.6393 | -101.5233 | D91 | MF125001 | MF124938 |
| 31 | 54.6393 | -101.5233 | D92 | MF125000 | MF124934 |
| 31 | 54.6393 | -101.5233 | D93 | - | MF124932 |
| 32 | 54.6381 | -101.5238 | D94 | - | - |
| 32 | 54.6381 | -101.5238 | D95 | MF124973 | - |
| 32 | 54.6381 | -101.5238 | D96 | MF125002 | MF124904 |
| 33 | 54.6294 | -101.5527 | D97 | MF124971 | MF124930 |
| 33 | 54.6294 | -101.5527 | D98 | MF124997 | MF124929 |
| 33 | 54.6294 | -101.5527 | D99 | - | MF124928 |

Appendix S1 continued.

|  | Latitude (Decimal degrees) | Longitude (Decimal degrees) |  | GenBank Accession Number | |
| --- | --- | --- | --- | --- | --- |
| Collection Site |  |  | Sample | ITS | Actin |
| 34 | 54.6296 | -101.5514 | D100 | - | MF124902 |
| 34 | 54.6296 | -101.5514 | D101 | MF124996 | - |
| 34 | 54.6296 | -101.5514 | D102 | MF124995 | - |
| P3 | 54.6514 | -101.5094 | P3 | KF317571.1 | KF317550.1 |
| P10 | 54.6478 | -101.5217 | P10 | KF317572.1 | KF317551.1 |
| P11 | 54.6467 | -101.5242 | P11 | KF317573.1 | - |
| P12 | 54.6411 | -101.5381 | P12 | KF317574.1 | - |
| P13 | 54.6333 | -101.5406 | P13 | KF317575.1 | - |
| P19 | 54.6456 | -101.5022 | P19 | JX645008.1 | KF317552.1 |
| P22 | 54.6406 | -101.5147 | P22 | KF317576.1 | - |
| P22b | 54.6406 | -101.5147 | P22b | JX645017.1 | - |
| P23 | 54.6411 | -101.5167 | P23 | JX645018.1 | KF317553.1 |
| P24 | 54.6417 | -101.5203 | P24 | KF317577.1 | - |
| P25 | 54.6467 | -101.5097 | P25 | KF317578.1 | - |
| P27 | 54.6294 | -101.5528 | P27 | KF317579.1 | - |
| P28 | 54.6475 | -101.5336 | P28 | JX645015.1 | KF317554.1 |
| MC2 | 54.6286 | -101.5533 | MC2 | KF317580.1 | - |


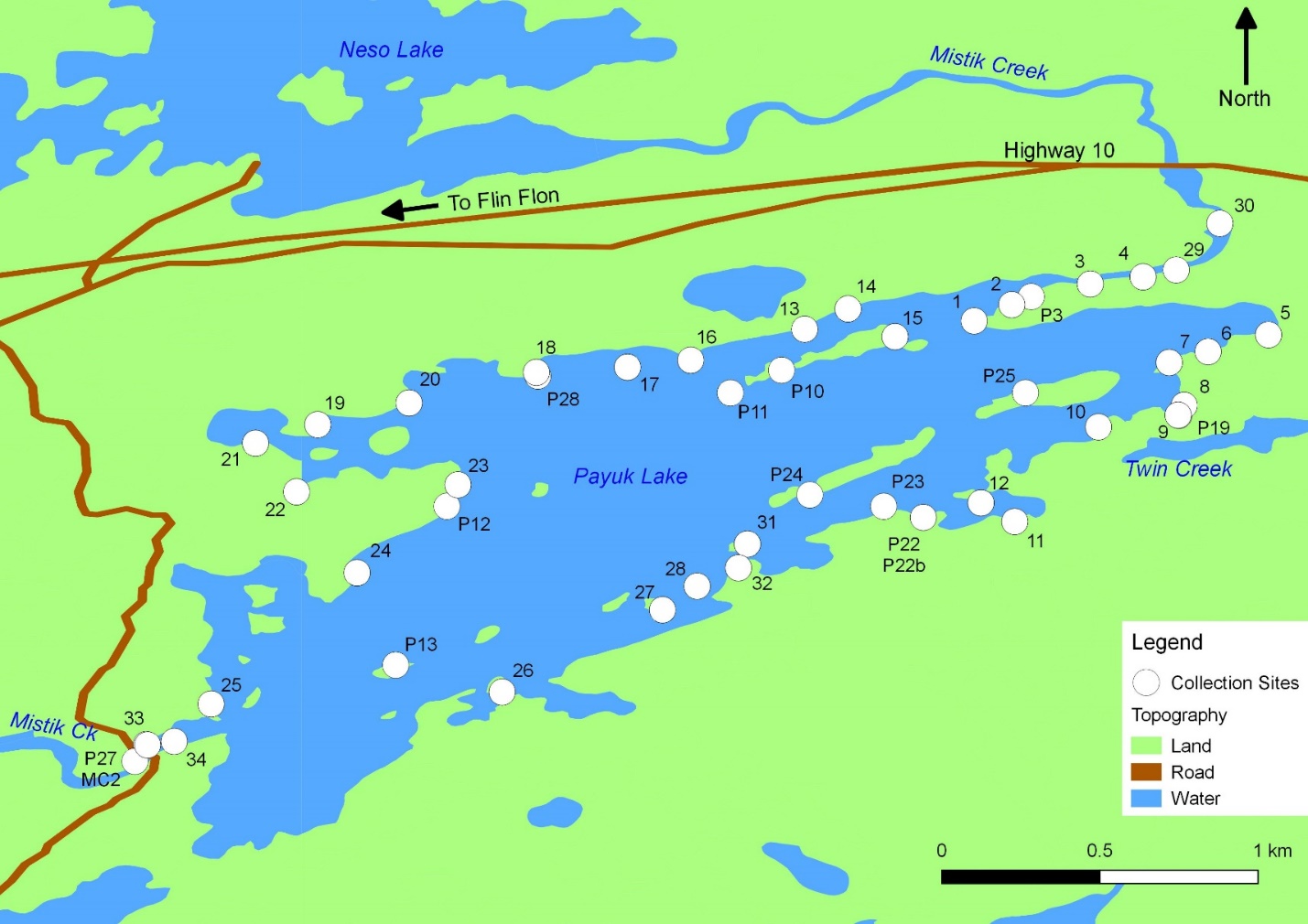


**Fig. S1.1** Collection sites of *Dermatocarpon luridum* (and therefore the photobiont *D. chodatii*) thalli from Payuk Lake, Manitoba, Canada. Numbers without letters represent samples collected in this study. Numbers with letters represent samples collected in Fontaine et al. (2013) where P is Payuk, MC is Mistik Creek. Each number in this study represents collections in triplicate. Map was produced using Quantum GIS v. 2.18 Las Palmas (Quantum GIS Development Team 2017), with base maps downloaded from CanVec (GeoGratis, Natural Resources Canada)

Table S2. Isolated algae from rock scrapings taken around thalli of *Dermatocarpon luridum* collected from Payuk Lake, Manitoba, Canada.

| Algal ID | Phylum | Colony | Shape | Size | Chloroplast Location | Mucilage | Other Characteristics |
| --- | --- | --- | --- | --- | --- | --- | --- |
| *D. chodatii* | Chlorophyta | Uni-cellular | Ellipsoidal- spherical | 3-5μm × 4-8μm | Parietal | No |  |
| *Diplosphaera*-like | Chlorophyta | Uni-cellular | Ellipsoidal | 3μm × 5μm | Parietal | No |  |
| *Trebouxia*-like | Chlorophyta | Uni-cellular | Spherical | 8.7μm × 9.1μm (single cell), 16.8μm × 16.8μm (with spores) | Central | Yes | Spores inside (4.9μm × 5.2μm) |
| *Coccomyxa*-like | Chlorophyta | Multiple unicells in mucilage | Spherical | 3.5μm × 3.9μm (cell), 7.1μm × 7.7μm (with mucilage) | Central | Yes |  |
| *Chlorella*-like | Chlorophyta | Uni-cellular | Spherical | 11.2μm × 12.1μm | Evenly interspersed | No | Large central vacuole |
| Unknown Chlorophyte 1 | Chlorophyta | Uni-cellular | Spherical | 5.4μm × 5.5μm | Central | No | Granular bodies present |
| Unknown Chlorophyte 2 | Chlorophyta | Uni-cellular | Spherical | 4.0μm × 4.4μm | Evenly interspersed | No |  |
| Unknown Cyanobacterium | Cyanophyta | Filamentous, single trichomes | Spherical | < 3μm | N/A | Yes | Heterocysts present |

**
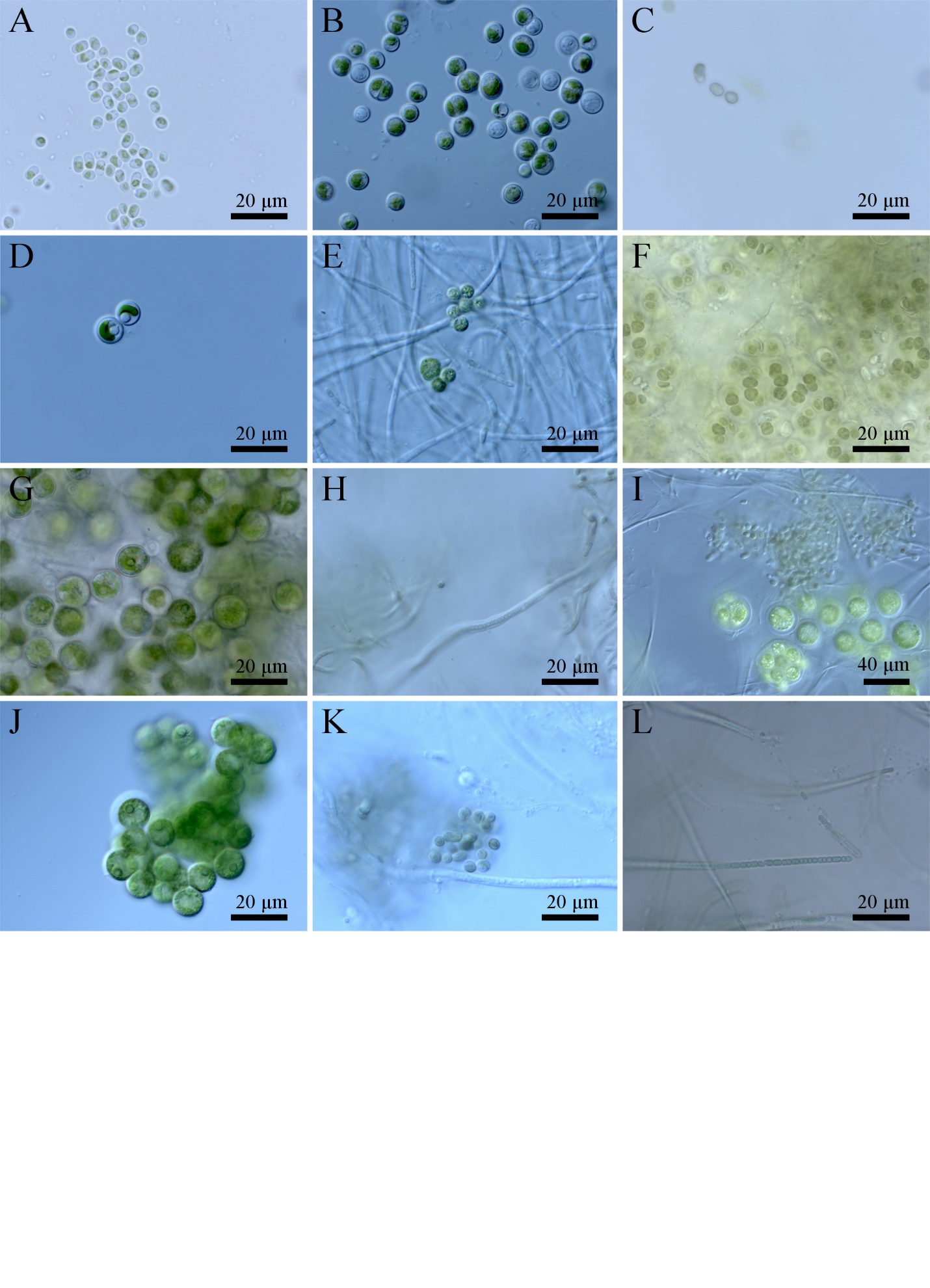
**

**Fig S1.2** Bright field (BF) and differential interference contrast (DIC) of isolated lichenised and associated algae from *Dermatocarpon luridum* from Payuk Lake, MB, Canada. A-C: Isolated *Diplosphaera chodatii*; A: axenic known culture (100x BF); B: lichenised (100x DIC); C: *D. chodatii*-like alga from rock scrapings surrounding *D. luridum* (100x, BF). D-H: Isolated algae associated with *D. luridum* thallus; D: *D. chodatii*-like alga (100x DIC); E: unknown Chlorophyte 1 (100x DIC); F: *Coccomyxa*-like green alga (100x BF); G: *Chlorella*-like green alga (100x BF); H: unknown cyanobacterium, lacking chloroplasts (100x BF). I-L: Isolated from rock scrapings surrounding *D. luridum*; I: *Trebouxia*-like green alga (40x DIC); J: *Chlorella*-like green alga (100x DIC); K: unknown Chlorophyte 2 (100x DIC); L: unknown cyanobacterium (100x BF). Photo credits: Kyle Fontaine.


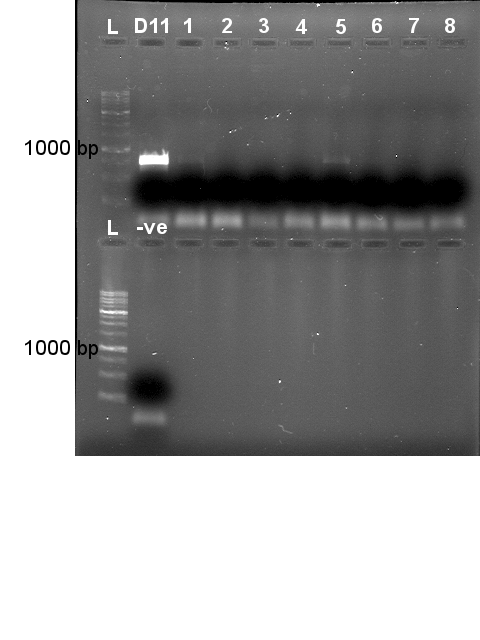


**Fig S1.3** Gel image of a 1% agarose gel showing the amplified product of the internal transcribed spacer region (ITS) of a lichenised (D11) and environmental sample of *Diplosphaera chodatii* collected from Payuk Lake, Manitoba. L: 1kb DNA ladder; -ve is the negative control. Lanes 1-4 are a dilution series (1:10, 1:100, 1:1000, and 1:10000 respectively) of environmental sample E24, taken from rock scrapings surrounding a *Dermatocarpon luridum* thallus. Lanes 5-8 are a dilution series (1:10, 1:100, 1:1000, and 1:10000 respectively) of environmental sample E35.
